# Systemic light chain cardiac amyloidosis with atrioventricular block

**DOI:** 10.3389/fcvm.2025.1573007

**Published:** 2026-01-08

**Authors:** Jiadan Liao, Xiaohong Pan

**Affiliations:** 1 Department of Cardiology, The Third Affiliated Hospital of Zhejiang Chinese Medical University, Hangzhou, China; 2 Department of Cardiology, Second Affiliated Hospital, College of Medicine, Zhejiang University, Hangzhou, China

**Keywords:** atrioventricular block, cardiac amyloidosis, implantable cardiac monitor, light chain type, systemic

## Abstract

**Background:**

Systemic light-chain (AL) amyloidosis is a rare plasma cell disorder with an annual incidence of approximately 8–12 cases per million population in regions with advanced diagnostic capabilities. Cardiac involvement occurs in 60%–75% of patients, and without treatment, the median survival is only six months. Atrioventricular (AV) conduction block affects approximately 3%–5% of AL patients. Although current therapies can induce deep hematologic remission and often significantly reverse myocardial structural and functional abnormalities, residual amyloid deposits may still trigger delayed, life-threatening conduction system events.

**Illustrative case:**

We describe a representative 50-year-old male patient with AL cardiac amyloidosis who achieved deep hematologic remission after standard anti-plasma cell therapy and autologous stem cell transplantation. Echocardiography demonstrated marked improvement in both myocardial structure and systolic/diastolic function. Nevertheless, he presented with syncope. An implantable cardiac monitor (ICM) captured a 7-second episode of asystole. A permanent pacemaker was subsequently implanted, resulting in complete resolution of syncope. Notably, atrial arrhythmias markedly decreased thereafter, and no episodes of paroxysmal atrial fibrillation have been documented on serial ambulatory electrocardiography or remote ICM monitoring during follow-up.

**Conclusions:**

This case highlights a crucial principle in the management of AL amyloidosis: improvement in hematologic status and cardiac structure/function does not equate to electrophysiologic stability. In high-risk patients, vigilance for occult conduction disease must persist even after clinical recovery. The ICM serves as a pivotal “bridge” tool to detect intermittent, life-threatening bradyarrhythmias and guide timely, precise device-based intervention. These findings underscore the need for individualized, long-term cardiac electrophysiological monitoring strategies in patients with AL-CA.

## 1 Introduction

Systemic light-chain (AL) amyloidosis is caused by the misfolding of monoclonal immunoglobulin light chains into amyloid fibrils that deposit in multiple organs. In regions with advanced diagnostic capabilities, its annual incidence is estimated at approximately 8–12 cases per million population (1). The heart is the most commonly involved organ and the primary determinant of prognosis; cardiac involvement occurs in 60%–75% of patients—a figure consistently reported across diverse populations, including North American, European, and Asian cohorts (2). Although atrial fibrillation is more prevalent, atrioventricular (AV) conduction block, though relatively rare (occurring in about 3%–5% of cases), represents a serious and potentially life-threatening complication that has been documented across multiple geographic regions (3).

## 2 Pathophysiological mechanisms of cardiac involvement and conduction system disease

Cardiac injury in AL amyloidosis results from the deposition of monoclonal light chains as rigid amyloid fibrils within the myocardial interstitium, leading to increased myocardial stiffness, diastolic dysfunction, and direct cardiomyocyte toxicity (4). The cardiac conduction system—comprising the sinoatrial node, atrioventricular node, and His-Purkinje network—is particularly vulnerable to amyloid infiltration and compression due to its small size and rich vascular supply. Histopathological studies have confirmed that amyloid deposits can encase and disrupt these critical structures, thereby interfering with electrical impulse propagation and causing progressive conduction delay or even complete heart block (5). Importantly, once this electrophysiological damage is established, it may persist or even progress despite effective anti-plasma cell therapy, as the clearance of amyloid matrix from the myocardium is an exceedingly slow process (6).

## 3 Diagnosis

AL Cardiac Amyloidosis (AL-CA) typically has an insidious onset, and early symptoms—such as fatigue, weight loss, and exertional dyspnea—are nonspecific, often leading to misdiagnosis as hypertrophic cardiomyopathy or heart failure. Diagnosis of AL-CA requires a multimodal, integrated approach. Initial suspicion should be high in patients presenting with “left ventricular hypertrophy with low electrocardiographic voltage”—a classic “voltage–mass mismatch.” Serum free light chain (FLC) assay is the cornerstone of screening, with an abnormal *κ* : *λ* ratio demonstrating sensitivity exceeding 95%. Echocardiographic assessment of global longitudinal strain (GLS) showing the “apical sparing pattern” is a highly specific imaging hallmark (7). Definitive diagnosis involves two key steps: First, confirm the presence of a monoclonal plasma cell disorder through serum free light chain assay, serum and urine immunofixation electrophoresis (IFE), and bone marrow aspiration and biopsy (to assess plasma cell percentage and clonality). Then confirmation of amyloid type and cardiac involvement: In patients with a typical cardiac phenotype, if ^99m^Tc-DPD/PYP scintigraphy shows grade 0–1 myocardial uptake and a monoclonal light chain is present, a diagnosis of AL cardiac amyloidosis can be established without endomyocardial biopsy. However, if scintigraphy demonstrates grade 2–3 uptake or diagnostic uncertainty persists, endomyocardial biopsy—with Congo red staining positive for amyloid deposits and mass spectrometry confirming immunoglobulin light-chain (AL) type—is required as the gold standard (8). This diagnostic algorithm not only enables accurate diagnosis of AL cardiac amyloidosis but also effectively differentiates it from transthyretin amyloidosis (ATTR) (Table 1) and multiple myeloma.

**Table 1:** Key differences between AL and ATTR cardiac amyloidosis.

Feature	AL amyloidosis	ATTR amyloidosis
Pathogenesis	Monoclonal immunoglobulin light chains (*κ* or *λ*) from clonal plasma cells	Misfolded transthyretin (TTR): wild-type (wtATTR) or mutant (hereditary)
Typical age at diagnosis	50–65 years	wtATTR: >70 years; hereditary: 50–80 years
Cardiac phenotype	Rapidly progressive restrictive cardiomyopathy; high early mortality	Slower progression; wtATTR predominantly affects elderly men
Extracardiac manifestations	Nephrotic syndrome, autonomic/sensory neuropathy, macroglossia, periorbital purpura	Hereditary: polyneuropathy; wtATTR: carpal tunnel syndrome, lumbar stenosis, biceps tendon rupture
ECG	Low QRS voltage in limb leads despite LV hypertrophy (“voltage–mass mismatch”)	Normal or high QRS voltage relative to wall thickness
Echocardiography	Concentric LVH, granular sparkling myocardium, apical-sparing GLS pattern (“cherry on top”)	LVH with less sparkling; apical sparing less consistent
Bone scintigraphy (⁹⁹^m^Tc-PYP/DPD/HMDP)	Negative or mild uptake (Grade 0–1)	Moderate–intense uptake (Grade 2–3) + negative monoclonal protein studies = diagnostic for ATTR
Serum biomarkers	↑ NT-proBNP, ↑ troponin; abnormal FLC ratio, ↑ dFLC	↑ NT-proBNP, ↑ troponin; normal FLC and serum immunofixation
Diagnostic gold standard	Tissue biopsy (Congo red+) + mass spectrometry: light chain–positive, TTR-negative	Biopsy + TTR-positive; or non-biopsy diagnosis per consensus criteria
First-line therapy	Daratumumab-based regimens (e.g., DARA-CyBorD); bortezomib; ASCT (selected)	Tafamidis; patisiran, inotersen, vutrisiran, eplontersen
Median Survival (untreated, cardiac)	<6 months (Mayo Stage III–IV)	wtATTR: ∼3–5 years after HF diagnosis
Critical clinical pitfall	Misdiagnosed as HFpEF or hypertensive heart disease	Misdiagnosis as AL leading to inappropriate chemotherapy

AL, light-chain amyloidosis; ATTR, transthyretin amyloidosis; LVH, left ventricular hypertrophy; GLS, global longitudinal strain; FLC, free light chain; dFLC, difference between involved and uninvolved FLC; HFpEF, heart failure with preserved ejection fraction.

## 4 Treatment and prognosis

The first-line treatment for AL-CA is daratumumab combined with cyclophosphamide, bortezomib, and dexamethasone (DARA-CyBorD), which efficiently induces deep hematologic response—defined as a difference between involved and uninvolved free light chains (dFLC) <40 mg/L and negative immunofixation electrophoresis—and significantly improves organ function [evidenced by reductions in cardiac biomarkers and imaging improvements such as decreased ventricular wall thickness and enhanced global longitudinal strain (GLS)] as well as overall survival (9). For selected patients aged ≤70 years with relatively preserved cardiac function (typically NT-proBNP <5,000 pg/mL and systolic blood pressure >90 mmHg), absence of severe autonomic dysfunction, and favorable multidisciplinary assessment, autologous stem cell transplantation (ASCT) may further deepen the response and prolong long-term survival; however, stringent patient selection is essential to minimize treatment-related mortality (10).

Regarding arrhythmia management: permanent pacemaker implantation is clearly indicated in patients with symptomatic high-grade (Mobitz type II second-degree) or third-degree atrioventricular (AV) block. For those experiencing life-threatening ventricular arrhythmias—such as sustained ventricular tachycardia, torsades de pointes, or ventricular fibrillation—an implantable cardioverter-defibrillator (ICD) may be considered if the expected survival exceeds 1 year and left ventricular ejection fraction (LVEF) is severely reduced (typically <35%) (11).

Risk stratification is best performed using the Mayo Clinic 2004/2012 staging system, which incorporates cardiac troponin T (cTnT), NT-proBNP, and dFLC to classify patients into stages I–IV, with median overall survival ranging from >90 months in stage I to <6 months in stage IV (12). Early diagnosis, prompt initiation of targeted therapy, and achievement of deep hematologic response represent the three pivotal determinants of improved long-term outcomes. Although these advances have substantially improved survival, they may also create a misleading impression that the disease is “fully controlled.” A critical clinical reality remains: amyloid infiltration of the cardiac conduction system—including the sinoatrial node, atrioventricular node, and His-Purkinje network—can cause irreversible structural damage. Even after pathogenic light chains are effectively eliminated, pre-existing amyloid deposits are cleared extremely slowly, leaving patients vulnerable to delayed, life-threatening arrhythmias such as high-grade AV block or prolonged sinus pauses. These events are often paroxysmal and unpredictable, making them difficult to capture with conventional electrocardiographic monitoring. Consequently, in the comprehensive management of AL-CA, improvement in hematologic status and cardiac structure/function should never be equated with electrophysiologic safety. For patients in remission who present with unexplained syncope or palpitations, effective outpatient arrhythmia monitoring poses a significant challenge—one where the implantable cardiac monitor (ICM) is proving to be uniquely valuable.

## 5 Case presentation

This article reports a representative case of systemic light-chain cardiac amyloidosis in which the patient, despite achieving deep hematologic remission following standardized anti-plasma cell therapy and autologous stem cell transplantation, experienced syncope due to a 7-s episode of asystole. A permanent pacemaker was ultimately implanted based on evidence provided by an ICM.

**Figure 1:**
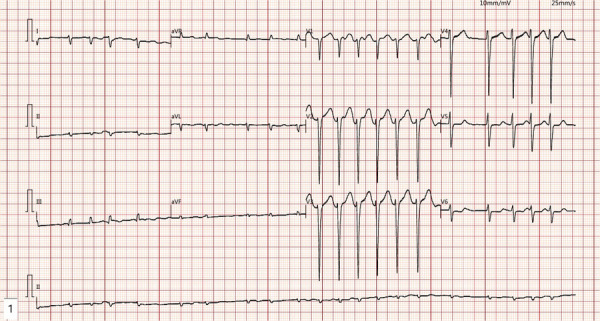
The ECG demonstrates rapid atrial fibrillation with a fast ventricular rate and low voltage in the limb leads.

**Figure 2:**
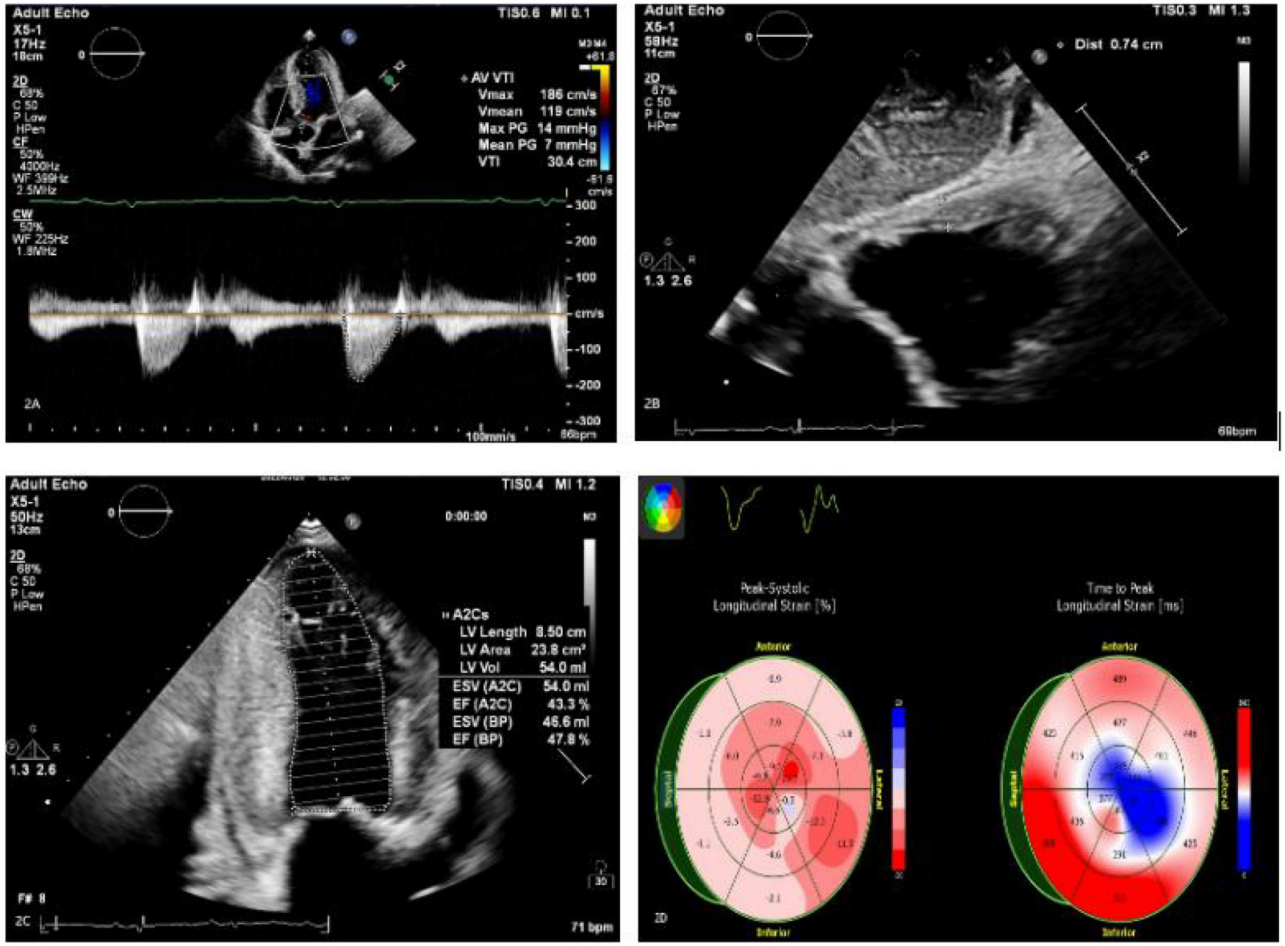
**(A–C)** Echocardiography findings include an enlarged left atrium and symmetrical thickening of the left ventricular myocardium (LVPWd 1.48 cm). Dense, granular sparkling echoes in the myocardium are observed, along with thickened right ventricular walls (free wall thickness 0.74 cm). Both mitral and tricuspid valves are thickened with mild regurgitation. There is reduced left ventricular systolic function (EF: 47.8%) and severely reduced left ventricular diastolic function (grade 3). A small amount of pericardial effusion is present. **(D)** The left ventricle shows preserved apical strain, presenting as a “cherry on top” sign. The left ventricular global longitudinal strain (LV GLS) is −6.2% (Left Ventricular Global Longitudinal Strain is a sensitive parameter for assessing left ventricular myocardial systolic function, derived from speckle-tracking echocardiography).

**Figure 3:**
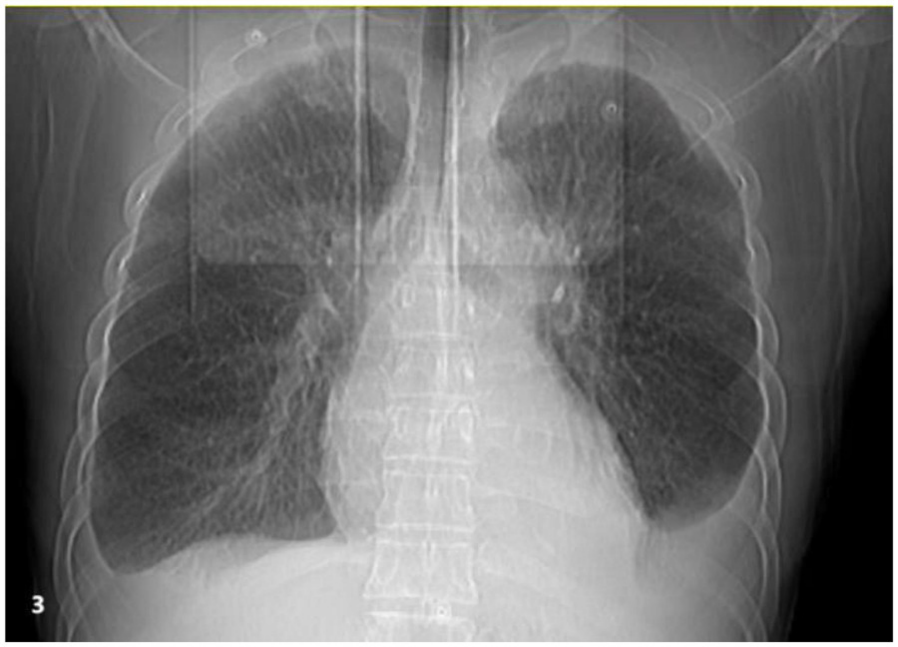
Chest imaging reveals bilateral moderate pleural effusions with atelectasis of the adjacent lung tissue caused by the pleural effusion.

The patient is a 50-year-old male who was admitted to the hospital on March 28, 2022, due to “chest tightness and dyspnea for 3 weeks after physical activity.” Physical examination revealed BP: 100/60 mmHg, HR: 150 beats per min, with an absolutely irregular heart rhythm, and oxygen saturation of 100%. The cardiac dullness border was enlarged; no jugular venous distension was observed, and pitting edema was present in both lower limbs. Muscle strength in all four limbs was normal.An electrocardiogram (ECG) (Figure 1) showed rapid atrial fibrillation with low voltage in limb leads. Echocardiography (Figure 2) suggested a primary consideration of cardiac amyloidosis: enlarged left atrium, symmetrical thickening of the left ventricular myocardium, dense and enhanced myocardial echoes with granular sparkling strong echoes, thickened right ventricular wall (free wall thickness 0.74 cm), preserved apical strain in the left ventricle presenting a “cherry on top” sign (LV GLS = −6.2%), thickened mitral and tricuspid valves with mild regurgitation, reduced left ventricular systolic function (EF: 47.8%), decreased left ventricular diastolic function (grade 3), and a small amount of pericardial effusion. The aortic valve had a congenital bicuspid malformation (Type 1), with currently acceptable valve function. Chest CT revealed moderate pleural effusion bilaterally with adjacent lung tissue atelectasis (Figure 3). Blood tests showed NT-proBNP: 5,150 pg/mL (normal: <125 pg/mL below75 years old), BNP: 650.4 pg/mL (normal: <100 pg/mL), LDH: 253 U/L (normal: 125–220 U/L), CK: 73 U/L (normal:38–174 U/L), CK-MB: 20 U/L (normal: <5–25 U/L), cTnI: 0.048 ng/mL (normal: <0.04 ng/mL).

**Figure 4:**
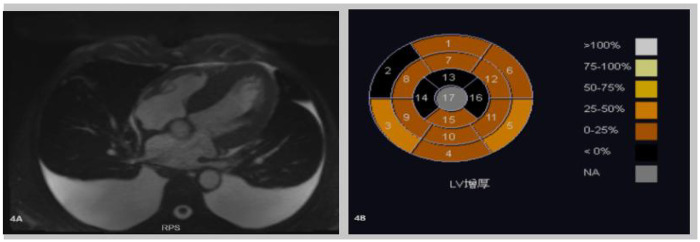
**(A,B)** Cardiac-enhanced MRI illustrates thickening of the left ventricular myocardium. Abnormal perfusion and enhancement changes in the right ventricular myocardium indicate myocardial amyloidosis. A small amount of pericardial effusion is also noted.

Cardiac enhanced MRI (Figure 4) demonstrated thickening of the left ventricular myocardium with abnormal perfusion and enhancement changes in the right ventricular myocardium, indicative of myocardial amyloidosis. A small amount of pericardial effusion was also noted, along with slight regurgitation of the mitral and tricuspid valves during systole.

Serum-free light chain assay: Serum free light chain assay revealed a markedly elevated lambda light chain (963.85 mg/L; normal: 5.7–26.3 mg/L), normal kappa light chain (10.32 mg/L; normal: 3.3–19.4 mg/L), and an abnormally low kappa/lambda ratio of 0.0107 (normal: 0.26–1.65), consistent with monoclonal lambda light chain production in AL amyloidosis.

Bone marrow aspiration was performed to determine the presence of clonal plasma cell proliferation and to confirm or rule out multiple myeloma or other plasma cell disorders. Myocardial biopsy was further conducted to confirm cardiac involvement and to differentiate the type of amyloidosis.

**Figure 5:**
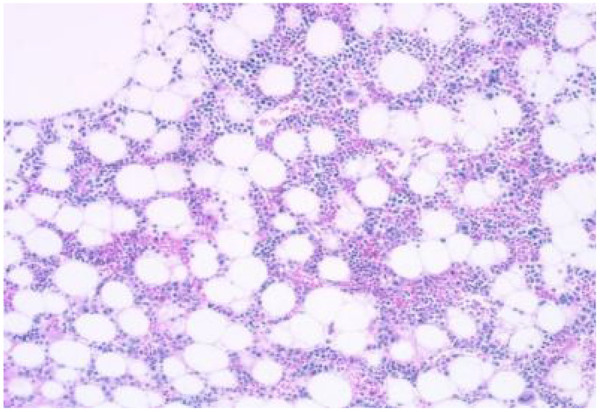
Bone marrow aspiration from the posterior superior iliac spine demonstrates active bone marrow proliferation with a red-to-yellow marrow ratio of 1:2. Infiltration of a few plasmacytoid cells within the bone marrow cavity is observed.

Bone marrow aspiration indicated active bone marrow proliferation in the posterior superior iliac spine, with a red-to-yellow marrow ratio of 1:2. There was an infiltration of a few plasmacytoid cells within the bone marrow cavity. Immunohistochemistry results showed clonal plasma cell expression, suggestive of a plasma cell neoplasm. CD235a+, CD123−, scattered CD20+, CD3−, MPO++, megakaryocytes CD42b+, CD71+, CD99−, CD34−, increased CD38 expression, increased CD138 expression, Kappa-, Lambda+, Ki-67 15%+, CD79a**+** (Figure 5).

**Figure 6:**
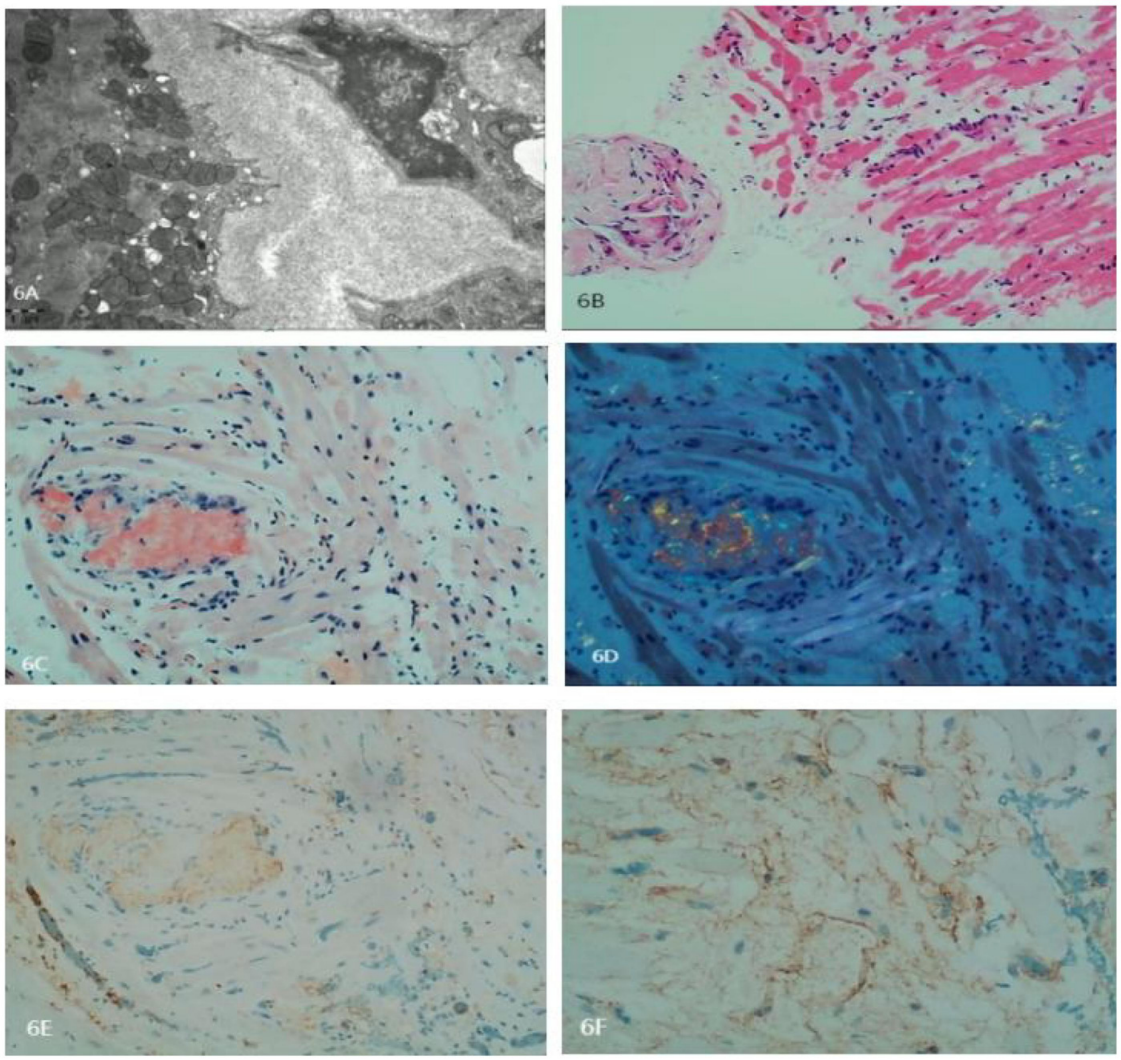
**(A)** Electron microscopy depicts degeneration and partial atrophy of myocardial cells. Myofibrils appear disorganized with focal degeneration. Extensive deposition of amyloid fibers in the interstitial and vascular walls is noted. The fibers are rigid, unbranched, and have diameters of approximately 8–12 nm, arranged in a disorganized manner. **(B)** Histopathology of the myocardial biopsy shows degeneration and atrophy in some myocardial cells. Intercellular spaces are markedly widened, and the walls of small vessels are thickened. Amyloid material is focally deposited in the interstitial and vascular walls. A small number of lymphocytes are seen infiltrating the region. **(C)** Congo red staining is positive. **(D)** Oxidized Congo red staining is positive, with apple-green birefringence observed under polarized light. **(E)** Negative staining for Kappa light chains. **(F)** Positive staining for Lambda light chains. Amyloid fibers exhibit strong binding to colloidal gold particles.

**Figure 7:**
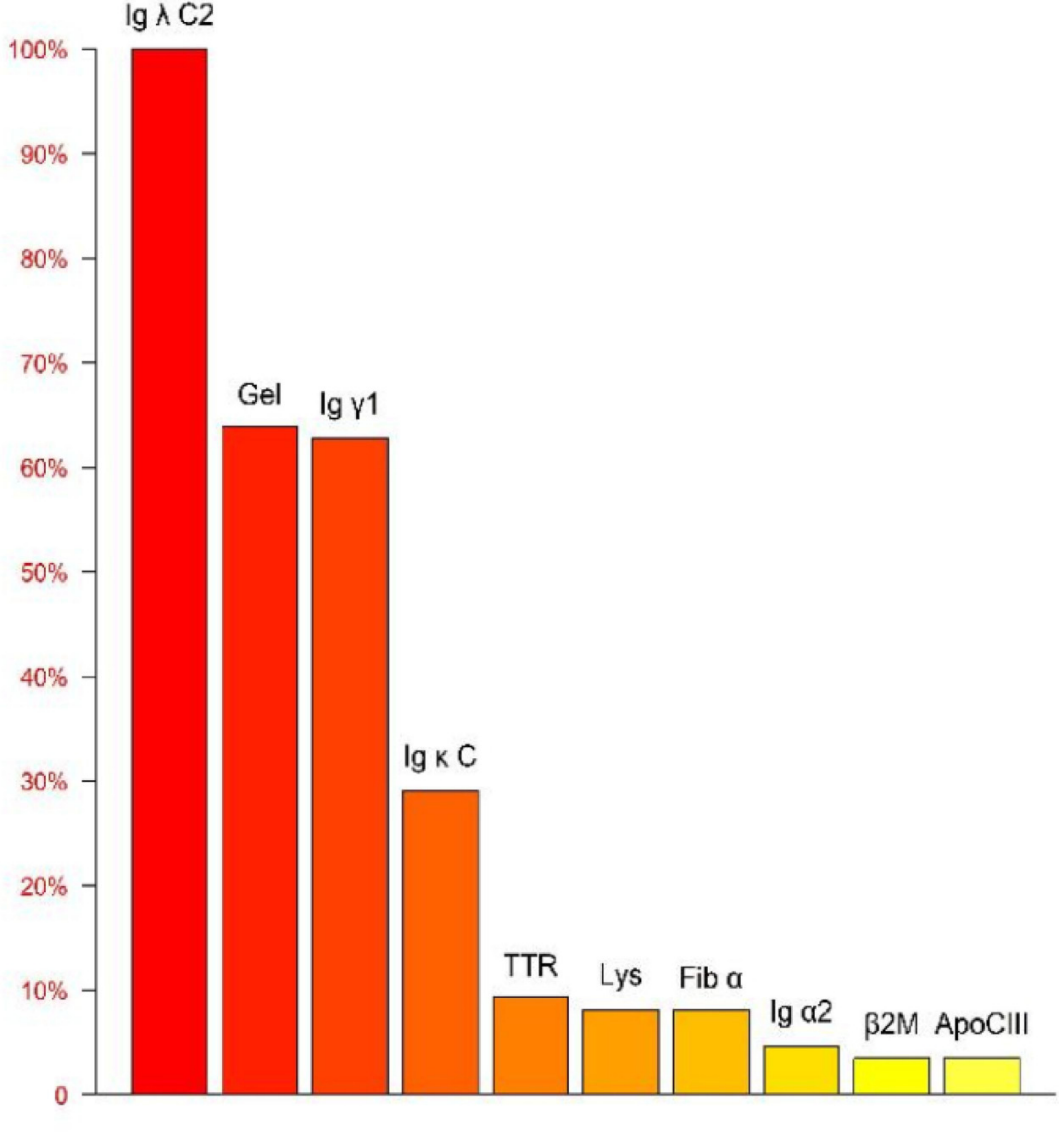
Mass spectrometry analysis of the amyloid protein identifies Ig *λ* as having the highest relative abundance, confirming the subtype as AL *λ* type.

Myocardial biopsy indicated AL-type amyloidosis. Special stains were Congo red positive and oxidized Congo red positive. Immunohistochemistry: Kappa-, Lambda+, AA− (Figure 6), indicating a classification of AL *λ* type (Figure 7).

Based on the patient's echocardiography, CMR, autoimmune immunoglobulin levels, serum free light chain levels, and histopathological examination results, the patient was diagnosed with AL-type cardiac amyloidosis.

**Figure 8:**
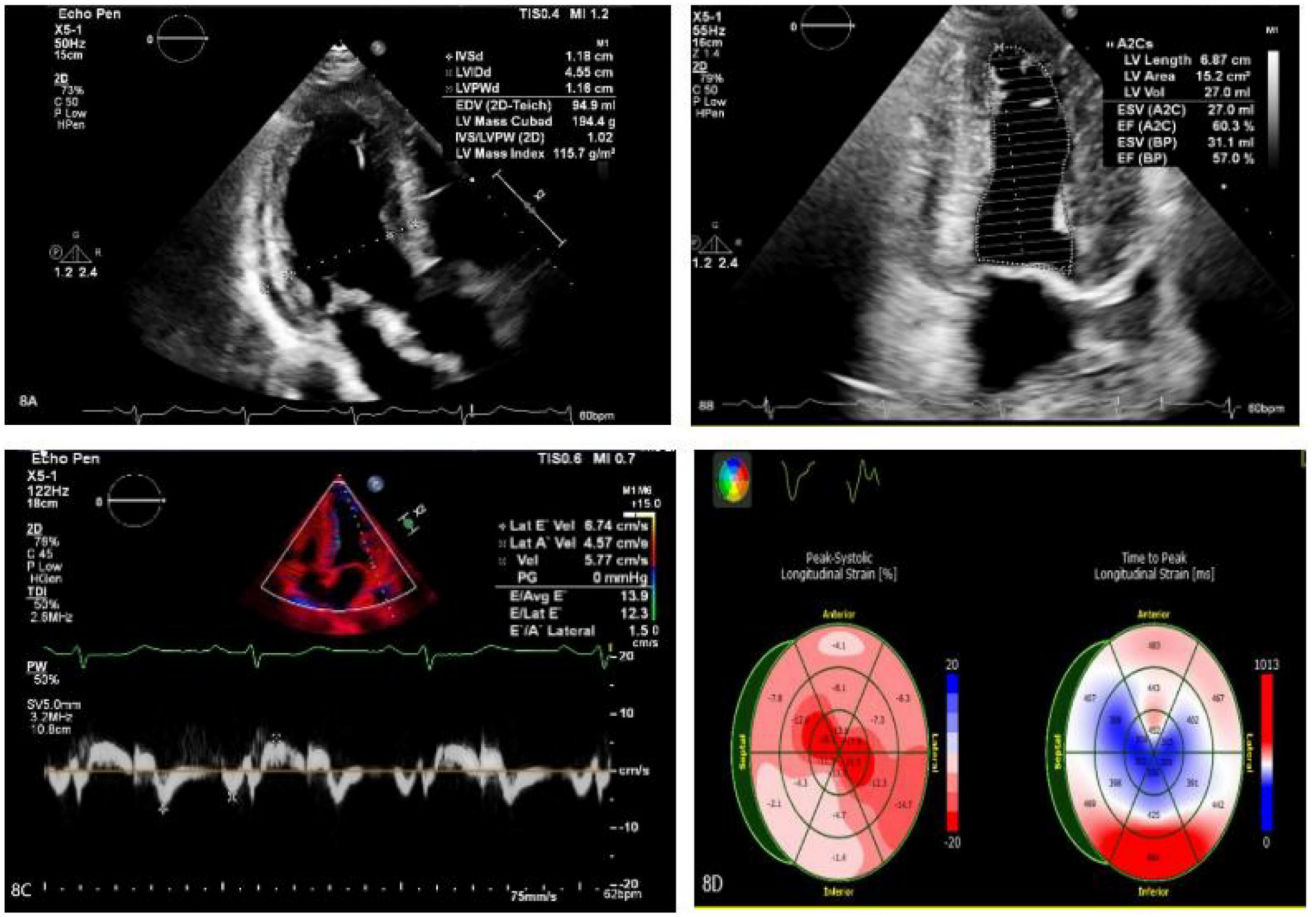
**(A–C)** The ventricular myocardium appears thinner compared to previous measurements: left ventricular posterior wall thickness (LVPWd): 1.16 cm; right ventricular free wall thickness: 0.45 cm. Left ventricular systolic function is normal (EF: 57%), with mildly reduced left ventricular diastolic function (Grade 1). **(D)** The left ventricular global longitudinal strain (LV GLS) is −10.4%. Apical strain is preserved, while basal and mid-segment strains are reduced.

**Figure 9:**
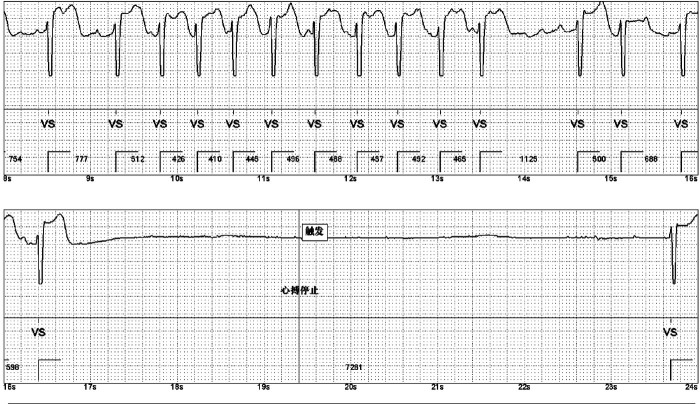
ICM indicates episodes of paroxysmal atrial fibrillation. A 7-second pause was recorded during one event.

**Figure 10:**
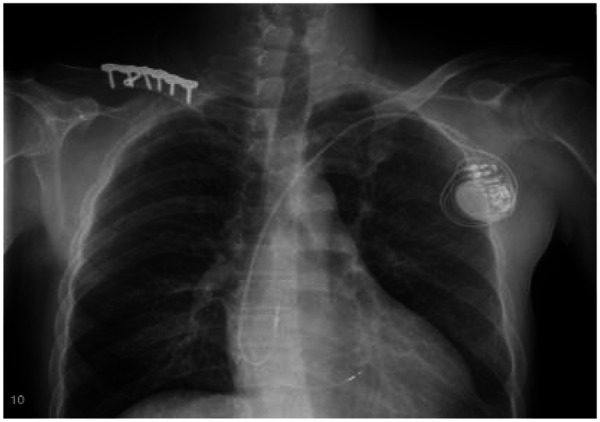
The patient underwent pacemaker implantation with left bundle branch pacing.

Treatment for the patient began on two weeks later. The first course of treatment using the VSD regimen (bortezomib 2.3 mg + cyclophosphamide 1.2 g + dexamethasone 40 mg) showed poor efficacy. The treatment was then changed to the DVCD regimen (bortezomib 2.3 mg + daratumumab 800 mg + cyclophosphamide 0.5 g + dexamethasone 40 mg). Due to persistently high heart rate after chemotherapy, the patient exhibited atrial flutter and paroxysmal atrial fibrillation on ECG, with a programmed AT/AF burden of 4%. Following chemotherapy, the patient developed persistent tachycardia accompanied by atrial flutter and paroxysmal atrial fibrillation. Despite receiving standard anticoagulation, electrical cardioversion, and diuretic therapy, serial follow-up visits revealed persistently elevated BNP levels. These findings suggested the possible presence of occult arrhythmias that had not been adequately captured or assessed by conventional electrocardiographic monitoring.Given the diagnosis of systemic AL-CA—a condition known to predispose the cardiac conduction system to infiltration by amyloid deposits—the patient was at significantly increased risk for developing advanced atrioventricular block, prolonged asystolic pauses, and even sudden cardiac death. However, at that time, there were no clear indications warranting immediate implantation of a permanent pacemaker or an implantable cardioverter-defibrillator (ICD). Therefore, to establish a definitive causal relationship between the patient's symptoms and potential arrhythmic events, and to enable precise risk stratification without premature device intervention, the clinical team decided to implement long-term, continuous outpatient cardiac rhythm monitoring. Accordingly, an implantable cardiac monitor (ICM) was implanted two months after the initiation of systemic therapy to objectively detect and evaluate potentially life-threatening bradyarrhythmias or tachyarrhythmias. One year later, the patient received HD-Mel conditioning (melphalan 150 mg on day 1, 100 mg on day 2, with supportive liver and stomach protection, antiemetic, prophylactic antimicrobial, and antithrombotic therapies) followed by autologous peripheral blood hematopoietic stem cell transplantation. Maintenance therapy with daratumumab was continued post-transplant. Subsequently, the patient's dFLC levels were normalized (1.35 mg/L) and stabilized. Echocardiography showed that the ventricular muscle had become thinner compared to before (LVPWd 1.16 cm, right ventricular free wall 0.45 cm), with left ventricular systolic function within the normal range (EF: 57%) and mildly reduced left ventricular diastolic function (grade 1), LV GLS = −10.4% (Figure 8). This year, the patient presented again with “dizziness and chest tightness for one week, accompanied by two episodes of syncope.” ICM interrogation revealed paroxysmal atrial fibrillation with a 7-s pause (Figure 9), prompting implantation of a permanent pacemaker (Figure 10). Since implantation of the dual-chamber pacemaker, the patient has not experienced any further episodes of syncope, and exercise tolerance during daily activities has significantly improved. We programmed the pacing rate at 80 beats per min. As of the time of manuscript submission, the patient had no recurrence of atrial fibrillation–related symptoms, and ambulatory electrocardiographic monitoring showed no frequent or rapid atrial arrhythmias.

## Discussion

Systemic AL-CA is a clonal plasma cell disorder caused by aberrant secretion of monoclonal immunoglobulin light chains from bone marrow plasma cells. These light chains misfold and aggregate into insoluble amyloid fibrils that deposit in the extracellular matrix of multiple organs (13). The heart is the most commonly involved organ and a key determinant of prognosis. Diagnosing AL-CA presents multiple challenges: First, amyloid deposition in the myocardial interstitium, microvascular walls, and conduction system leads to myocardial stiffness, diastolic dysfunction, microcirculatory impairment, and electrophysiological instability. Clinical manifestations—such as fatigue, exertional dyspnea, and edema—are nonspecific, often resulting in diagnostic delays. Second, although noninvasive tests like cardiac MRI can strongly suggest the diagnosis, they cannot replace histopathological typing. Current guidelines recommend that in patients with a typical cardiac phenotype, if serum free light chains (sFLC) are markedly abnormal (dFLC ≥18 mg/L) and bone marrow or abdominal fat pad biopsy is positive, endomyocardial biopsy may be avoided. However, when ⁹⁹^m^Tc-DPD/PYP scintigraphy shows grade 2–3 uptake or diagnostic uncertainty persists, myocardial biopsy (with Congo red staining and mass spectrometry) remains the gold standard. Third, it is essential to rigorously differentiate AL amyloidosis from ATTR amyloidosis, as their treatment strategies are fundamentally distinct.

The cornerstone of AL-CA management is rapid suppression of pathogenic light chain production (4). The first-line regimen is DARA-CyBorD (daratumumab plus cyclophosphamide, bortezomib, and dexamethasone), which significantly improves rates of hematologic and organ responses. Eligible patients—typically aged ≤70 years, with NT-proBNP <5,000 pg/mL, systolic blood pressure >90 mmHg, and no severe autonomic neuropathy—may proceed to autologous stem cell transplantation (ASCT) to enhance long-term survival. Notably, even after achieving deep hematologic remission (normalized dFLC and negative immunofixation), pre-existing amyloid deposits clear extremely slowly, and conduction system damage is often irreversible. Patients remain at risk for life-threatening arrhythmias—including sustained ventricular tachycardia (VT), ventricular fibrillation (VF), torsades de pointes, high-grade or third-degree atrioventricular block (AVB) with prolonged pauses, and sinus arrest. As illustrated in this case, despite deep remission following DARA-CyBorD and ASCT and significant improvement in cardiac structure and function, the patient experienced recurrent BNP elevation, persistent tachycardia, atrial arrhythmias, and a 7 s asystolic pause leading to syncope—highlighting the lag between structural/electrophysiological recovery and the potential irreversibility of some damage.

This case also highlights two major challenges in the management of AL-CA and underscores the importance of individualized decision-making regarding device therapy. First, conventional ECG or Holter monitoring frequently fails to capture intermittent arrhythmias occurring outside the hospital setting. Second, there is currently insufficient high-quality evidence to support routine prophylactic implantation of ICD or pacemakers in all patients. In this context, early implantation of an ICM represents a reasonable and individualized strategy for patients with unexplained syncope who lack clear indications for permanent pacing.The ICM enable continuous monitoring for up to three years, serving as a highly effective “diagnostic bridge” to capture symptom-correlated arrhythmias and provide definitive evidence to guide permanent device therapy. In this case, the ICM successfully documented prolonged sinus arrest, leading to timely implantation of a dual-chamber pacemaker and effectively reducing the risk of sudden death.

AL amyloidosis leads to diffuse myocardial infiltration, ventricular wall stiffening, and restrictive cardiomyopathy, which is a pathophysiological state that is highly sensitive to ventricular electromechanical synchrony. Conventional right ventricular apical pacing (RVP) often results in a non-physiological sequence of ventricular activation, causing significant electromechanical dyssynchrony that may further exacerbate diastolic dysfunction, reduce cardiac output, and accelerate the deterioration of heart function. In contrast, left bundle branch pacing (LBBP) directly captures the His-Purkinje conduction system, enabling a more physiologic pattern of ventricular depolarization and contraction. This approach effectively preserves ventricular synchrony and thereby mitigates the risk of pacing-induced ventricular remodeling. In this patient, clear evidence of myocardial involvement was already present prior to pacemaker implantation (LVEF was 47.8%, and GLS was markedly reduced at −6.2%), indicating borderline systolic function. Given the underlying disease process, which commonly causes conduction system infiltration and predicts a high burden of future ventricular pacing, the team opted for LBBP during the procedure. This strategic decision aimed to ensure reliable pacing support while maximizing the preservation of ventricular synchrony, thereby preventing pacing-induced cardiomyopathy and optimizing long-term outcomes.Given the myocardial stiffness and severely impaired diastolic function caused by cardiac amyloidosis, the patient's stroke volume was markedly reduced, rendering cardiac output highly dependent on heart rate for compensation. Therefore, the pacemaker's lower rate limit was programmed to 80 beats per min to moderately increase heart rate, thereby maintaining adequate cardiac output and improving hemodynamic status. Additionally, this relatively higher pacing rate may have exerted an anti-arrhythmic effect, which potentially by suppressing atrial electrical instability and reducing long pauses, consistent with the concept of “pacing-mediated suppression of atrial fibrillation”. Post-implantation follow-up confirmed the absence of atrial fibrillation–related symptoms, and ambulatory electrocardiographic monitoring revealed no frequent or rapid atrial arrhythmias.

In summary, although AL-CA is rare, it progresses rapidly and carries a grave prognosis; without timely recognition and treatment, outcomes are extremely poor. Early identification of “red flags” (such as low-voltage ECG with left ventricular hypertrophy, carpal tunnel syndrome, or macroglossia), accurate subtyping, prompt initiation of targeted therapy, and individualized cardiac management, including judicious use of ICM surveillance are critical for improving prognosis and reduce the risk of sudden cardiac death. Although current anti-plasma cell therapies can induce deep hematologic remission and even reverse myocardial structural abnormalities, residual amyloid deposits within the cardiac conduction system may still lead to irreversible electrophysiological dysfunction. Even when clinical and biochemical parameters appear “completely normalized,” electrophysiological stability often lags significantly behind. Therefore, vigilance for conduction system events occurring outside the hospital setting remains essential—even in patients who have achieved deep remission and substantial cardiac recovery. Furthermore, in specific high-risk scenarios—such as patients with unexplained syncope, those with negative short-term electrocardiographic evaluations but high clinical suspicion of arrhythmia, individuals with Mayo Clinic stage III or higher disease, and patients who, despite achieving hematologic remission, exhibit fluctuating BNP levels or new-onset arrhythmic symptoms—the ICM has emerged as a pivotal “bridge” tool for long-term outpatient monitoring.
